# An RNAi *in silico *approach to find an optimal shRNA cocktail against HIV-1

**DOI:** 10.1186/1743-422X-7-369

**Published:** 2010-12-20

**Authors:** María C Méndez-Ortega, Silvia Restrepo, Luis M Rodríguez-R, Iván Pérez, Juan C Mendoza, Andrés P Martínez, Roberto Sierra, Gloria J Rey-Benito

**Affiliations:** 1 Grupo de Virología SRNL, Instituto Nacional de Salud, Avenida Calle 26 No. 51 - 20 ZONA 6 CAN, Bogotá, Colombia; 2 Laboratorio de Micología y Fitopatología de la Universidad de los Andes, Bogotá, Colombia; 3 Grupo ASIS, Instituto Nacional de Salud, Bogotá, Colombia; 4 VC@SOFT, Bogotá, Colombia

## Abstract

**Background:**

HIV-1 can be inhibited by RNA interference *in vitro *through the expression of short hairpin RNAs (shRNAs) that target conserved genome sequences. *In silico *shRNA design for HIV has lacked a detailed study of virus variability constituting a possible breaking point in a clinical setting. We designed shRNAs against HIV-1 considering the variability observed in naïve and drug-resistant isolates available at public databases.

**Methods:**

A Bioperl-based algorithm was developed to automatically scan multiple sequence alignments of HIV, while evaluating the possibility of identifying dominant and subdominant viral variants that could be used as efficient silencing molecules. Student t-test and Bonferroni Dunn correction test were used to assess statistical significance of our findings.

**Results:**

Our *in silico *approach identified the most common viral variants within highly conserved genome regions, with a calculated free energy of ≥ -6.6 kcal/mol. This is crucial for strand loading to RISC complex and for a predicted silencing efficiency score, which could be used in combination for achieving over 90% silencing. Resistant and naïve isolate variability revealed that the most frequent shRNA per region targets a maximum of 85% of viral sequences. Adding more divergent sequences maintained this percentage. Specific sequence features that have been found to be related with higher silencing efficiency were hardly accomplished in conserved regions, even when lower entropy values correlated with better scores. We identified a conserved region among most HIV-1 genomes, which meets as many sequence features for efficient silencing.

**Conclusions:**

HIV-1 variability is an obstacle to achieving absolute silencing using shRNAs designed against a consensus sequence, mainly because there are many functional viral variants. Our shRNA cocktail could be truly effective at silencing dominant and subdominant naïve viral variants. Additionally, resistant isolates might be targeted under specific antiretroviral selective pressure, but in both cases these should be tested exhaustively prior to clinical use.

## Background

Despite the advent of highly active antiretroviral therapy (HAART), human immunodeficiency virus (HIV-1) is still a matter of concern for public health [1]. The major obstacle to finding a cure lies in the integration of the viral genome, by virtue of which the virus will always have a chance to restart the infection [2]. The overwhelming genetic variability of HIV-1 is mainly due to the error-prone nature of reverse transcriptase (RT) [3]. Other factors are also responsible for generating quasispecies, and usually a combination of factors -genetic (e. g. HLA type), immunological (e. g. CD8+ cytotoxic T lymphocytes selective pressure) and viral (e. g. HIV type, subtype, recombination events) among others- contributes to the exhaustion of the immune system [4,5]. Moreover, the virus has an innate ability to accumulate mutations that are readily accepted by its flexible proteins [6]. Collectively, these factors help the virus to overcome HAART [7]. Clearly, effective strategies are needed to combat each replication-competent viral variant that may emerge under any circumstances or selective pressure [8,9]. Although HAART saves thousands of lives, resistant variants emerge, even though multiple key steps in the viral replication cycle are targeted simultaneously [10]. Indeed, some cases have shown persistent viral replication, even under successful HAART [11,12].

RNA interference (RNAi) is an evolutionarily conserved naturally occurring eukaryotic process by which double-stranded RNA (dsRNA) triggers post-transcriptional gene silencing [13]. Research during the last decade has focused on the possibility of using it to treat various diseases [14]. In fact, several *in vitro *and *in vivo *RNAi approaches have proven effective at inhibiting HIV-1 [15-17], and such studies have shown that replication is potently inhibited beyond initial replication only when multiple conserved regions in the viral genome are targeted simultaneously [18,19]. However, even though HIV-1 has been inhibited *in vivo *in a humanized mouse model [20], there is no absolute certainty this will extrapolate to humans. Key differences between mouse models and humans may influence the viral population and its evolution, especially if complete inhibition is not achieved.

shRNA design to date has been based on studies of HIV variability that have focused on conserved regions and multiple sequence alignments (MSAs) [21], in which the HXB2 reference genome has been used to select the consensus silencing sequence. Efficient silencing molecules have also been selected by *in vitro *screening [17]. Previous studies analyzing 170 and 495 full-length genomes identified 19 and 216 target sequences respectively, showing that a greater number of viral genomes provides more evidence for variability [18,22]. Other authors have analyzed the conservation of unique targets from gene sequence fragments of 19 nucleotides [23]. However, 75% conservation among its genomes still allows the virus 25% variability, which it can use to escape from shRNA-based silencing. This highlights the importance of analyzing not only previously reported parameters of silencing efficiency [24,25], but also enough sequences to represent the actual viral variability. We addressed this issue by including in our analysis resistant isolates and more than 1000 viral genomes representing the M group viral divergence. The principal target was RT, but we further analyzed complete genome sequences. *In silico *studies can produce accurate enough approximations to guide better experimental approaches; thus, with this in mind, we developed an *in silico *approach for identification of the best HIV silencing molecules. Our *in silico *approach scanned multiple HIV-1 aligned genomes in search for the most frequent (dominant and subdominant) nucleotide variants in several conserved regions instead of identifying a single consensus sequence for each, in order to be able to use them all simultaneously in a combination cocktail. These variants were analyzed following Zhou and Zeng's [24] parameters in order to select the ones that could be efficient shRNAs, given a silencing score and an exhaustive search for off-target effects.

## Results

### Conserved regions and prevalent drug-resistant mutations

**Figure 1:**
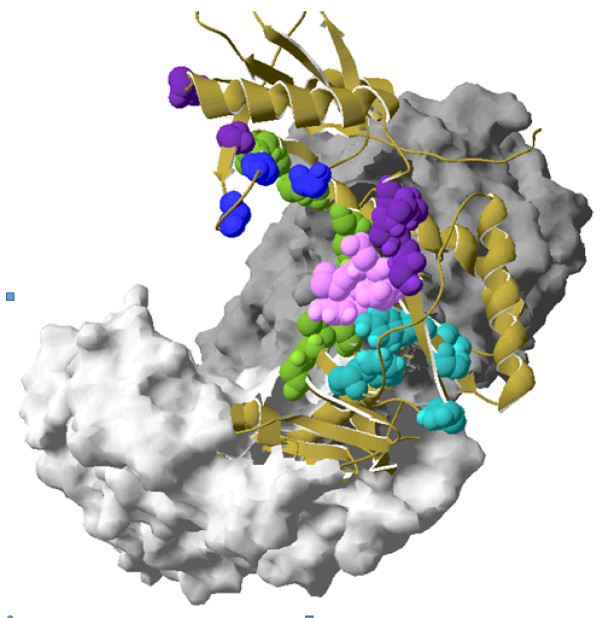
**Crystallographic structure of RT indicating Selected Regions**. **(a) **RT crystallographic structure 2ZD1 (1.8 Å) highlights the residues within the selected regions, Dark gray = p66 subunit, light gray = p55, dark blue = active site residues involved in dNTP binding (K65, R72, D110, V111, G112, D113, A114, Y115, Q151), green = active site residues involved in DNA binding (L74, V75, D76, R78, N81, E89, Q91, L92, I94, G152, K154, P157, M230, G231), purple = active site residues with no specific annotations (W24, P25, F61), pink = YMDD motif (Y183, M184, D185, D186), and light blue = residues involved in NNRTI binding (L100, K101, K102, K103, V179, Y188, G190, F227; not conserved). Ribbon shows continuity between amino acid chains.

The homology searches of the RT_Rtv cd01645 protein domain were used for a BLAST search against the reference HIV-1 POL protein in the NCBI Conserved Domain Database (CDD) with a cut-off of 1e-85. Within this domain, nine subregions were mapped that were associated with DNA binding sites, dNTP binding sites, reverse transcriptase inhibitor (RTI) binding sites, active site residues with no other annotations, and the motif YMDD (Figure 1). Highly prevalent drug-resistant mutations located within or adjacent to these regions were identified. Table 1 shows the selected regions with their wild type residues and drug-resistant substitutions with corresponding prevalence based on HIVRT&PrDB data. Positions were mapped with respect to the HXB2 reference genome sequence. All regions were analyzed for each MSA.

**Table 1:** Target regions within Conserved Domain RT_rtv

**Region No**.	Residue Position (wild type)	Residue (wild type)	Function annotation	HXB2 coordinates	Mutation in RT^2 ^and prevalence^3^	Evaluated Region in MA^4^
1	24, 25	**W,P**	DBS	2619-2622	-	2610 - 2630

2	60	V	-	2727	I(14)	2700 - 2760
	61	**F**	AS	2730	-	
	62	A	-	2733	V(14)	
	64	K	-	2736	R(1.9)	
	65	**K**	dBS	2742	R(2.1)	
	67	D	-	2748	N(38), G(2.5)	

3	72	**R**	dBS	2763	-	2750 -2800
	74-76, 78, 81	**L,V,D,R,N**	DBS-AS	2769-2790	-	

4	89	**E**	DBS-AS	2814	-	2800 - 2840
	90	V	-	2828	I(3.3)	
	91,92,94	**Q,L,I**	DBS-AS	2820-2829	-	

5	100-103	L,K,K,K	NNBS	2847-2856	-	2835 - 2870

6	110-115	**D,V,G,D,A,Y**	dBS-AS	2877-2892	-	2865 - 2905

7	151	**Q**	dBS-AS	3000	M(3.4)	2985-3020
	152,154,157	**G,K,P**	DBS-AS	3003-3018	-	

8	178	I	-	3081	M(7.5), L(6.4)	3070-3130
	179	V	NNBS	3084	I(7.9), D(1.3)	
	181	Y	-	3090	C(14)	
	183	**Y**	NNBS-DBS-AS	3096	-	
	184	M	variable	3099	V(50), I(1.4)	
	185	**D**	dBS-AS	3102	-	
	186	**D**	AS	3105	-	
	188	Y	NNBS	3111	L(3.5)	
	190	G	NNBS	3117	-	

9	227	**F**	NNBS	3228	L(1.6)	3210-3255
	228	L	-	3231	H(12), R(4.2)	
	230	**M**	DBS-AS	3237	L(1.8)	
	231	**G**	DBS-AS	3240	-	

^1^Positions according to the HXB2 reference genome numbering system (coordinate map)

^2^RT drug resistance mutations prevalence was calculated from 17,167 sequences exposed to either of these drug types (HIV Drug Resistance Database)

^3^Mutation prevalence (percent) data are available at the HIV Drug Resistance Database

^4^MSA: multiple alignment

In bold, residues directly involved in enzyme activity

DBS, DNA binding site

dBS, dNTP binding site

NNBS, non-nucleoside reverse transcriptase inhibitor binding site

AS, active site

### Sequence retrieval and MSAs

A total of 2,264 sequences from the non-specific first line regimen were downloaded from HIVRT&PrDB and aligned. In addition, four specific MSA from specific regimens were generated independently, but with caution on not including sequences with previous treatment history: Stavudine-Lamivudine-Nevirapine (D4T-3TC-NVP) MSA (91 sequences), Zidovudine-Lamivudine-Efavirenz (ZDV-3TC-EFV) MSA (1,381 sequences), Zidovudine-Lamivudine-Abacavir (ZDV-3TC-ABC) (52 sequences) and Zidovufine-Lamivudine-Nevirapine (ZDV-3TC-NVP) (212 sequences). Six MSA from Los Alamos HIV databases were used to assess the impact of viral diversity: three from only the *pol *gene (B subtype no recombinants, 778 sequences; Group M plus recombinants, 1206 sequences; all subtypes, 1250 sequences) and three from complete HIV genomes (B subtype no recombinants, 790 sequences; Group M plus recombinants, 1214 sequences; all subtypes, 1257 sequences). Some sequences were present in more than one MSA and were discarded.

Thirty-five Colombian samples from hospitalized symptomatic HIV-positive patients with viral loads over 1000 copies/ml were chosen for genotyping and were analyzed so that the sequences from resistant isolates could be included in the study (resistance data will be published separately). These isolates were added to the 2,264 resistant isolate alignment to give a 2299 sequence alignment. Accession numbers are: [GenBank:HM584982, GenBank:HM584983, GenBank:HM584984, GenBank:HM584985, GenBank:HM584986, GenBank:HM584987, GenBank:HM584988, GenBank:HM584989, GenBank:HM584990, GenBank:HM584991, GenBank:HM584992, GenBank:HM584993, GenBank:HM584994, GenBank:HM584995, GenBank:HM584996, GenBank:HM584997, GenBank:HM584998, GenBank:HM584999, GenBank:HM585000, GenBank:HM585001, GenBank:HM585002, GenBank:HM585003, GenBank:HM585004, GenBank:HM585005, GenBank:HM585006, GenBank:HM585007, GenBank:HM585008, GenBank:HM585009, GenBank:HM585010, GenBank:HM585011, GenBank:HM585012, GenBank:HM585013, GenBank:HM585014, GenBank:HM585015, GenBank:HM585016].

### Variability analysis and shRNA design

A total of 48 shRNAs were found that could be used for silencing HIV effectively based on the number of targeted sequences in each MSA -targeted sequences are those that matched the shRNA sequence- and the number of hits on more than one MSA (Additional file 1). From these we sort out a reduced number that could target the greatest number of sequences in order to optimize their use in gene therapy. All of these shRNAs fit the free energy criteria (≥-6.6 kcal/mol), which is thought to be the most important factor for silencing. Resistant isolates showed greater variability, which is consistent with the calculated entropy values obtained for each one. Table 2 shows the percentage of coverage of each set of frequent shRNAs for each MSA. These percentages were calculated as the number of sequences that matched the exact shRNA sequence with respect to the total amount of viral sequences included within each MSA. Given the different number of total viral sequences that were included in analyses, we used percentages in order to be able to compare results between different MSAs. The number of viral sequences included in the analyses (NSI) and the number of viral variants (VV) -the latter including dominant and subdominant viral variants-- together give an indirect measure of variability for each MSA in a specific window. The ideal window is that whereby the greatest number of sequences of a MSA could be included for the analyses, and that showed the least number of viral variants. Of course, this would demonstrate that part of the viral genome is not changing much and shows little worldwide diversity -represented by the online available worldwide sequences. Also the number of subdominant variants (SV) for each window is an initial measure of variability, for the perfect window should have the smallest number of viral variants able to target most of the sequences. This also happens with the number of sequences that might be targeted by the group of subdominant variants (ST-SV); this value indicates how many sequences might be silenced by perfect sequence matching and efficient silencing features, using the cocktail of shRNAs directed to all these subdominant variants. Regarding this variable, Table 2 shows that a cocktail of shRNAs based on targeting the subdominant variants might be able to target more than 90% of the sequences (column PC-SV). Comparing PC-SV that can reach up to 96% of sequences targeted, against PC-DV which reaches well under 80%, it can be said that a cocktail of shRNAs design based on subdominant variants has a higher chance of targeting more viruses. Table 3 shows the shRNAs that target sequences in more than one MSA. In each MSA a set of sequences were eliminated due to a high content of ambiguous bases in the analyzed window, or because they were repeated. The scores are the result of different sequence features that could improve silencing by enhancing the uploading of the guide strand into the silencing complex (Additional file 2). Table 3 shows the shRNAs capable of targeting several sequences in highly divergent MSAs, with the possibility of targeting more than one viral subtype and even recombinants. The first three pairs of coordinates have shRNAs that were identified in non-resistant MSAs and the last three have shRNAs that were identified in resistant MSAs. Scores are clearly different between both groups, and similar within each group. shRNAs from resistant isolates showed the lowest score values. As expected, the dominant viral variant -usually matching HXB2 reference genome-- virtually targeted the greatest amount of sequences. The others are virtually able to target other viral variants -subdominant and infrequent.

**Table 2:** MSA coverage by shRNAs

^a ^MSA	^b ^NSI	^c ^VV	^d ^W	^e ^E	^f ^SV	^g ^ST-SV	^h ^PC -SV (%)	^i ^ST-DV	^j ^PC-DV (%)
Pol Subtype B no Recombinants	747	35	1	1.36	11	712	95.31	588	78.71

Pol Group M plus Recombinants	1143	46	1	1.42	12	1088	95.18	913	79.88

Pol All Subtypes	1160	52	1	1.59	14	1102	95	916	78.97

Genome Subtype B no Recombinants	760	35	1	1.35	12	728	95.79	599	78.82

Genome Group M plus Recombinants	1153	46	1	1.41	12	1098	95.22	918	79.62

Genome All Subtypes	1169	52	1	1.60	13	1107	94.7	920	78.69

ZDV-3TC-EFV	1185	27	1	1.27	10	1169	98.65	1013	85.49
	
	1201	30	2	1.52	14	1177	98	926	77.1
	
	1348	53	3	1.94	13	1303	96.66	741	54.97

2299_resistant_isolates	1547	26	1	1.72	14	1552	98.84	1255	80.86

D4T-3TC-NVP	79	13	1	1.9	4	68	86.08	52	65.82

ZDV-3TC-ABC	52	10	1	1.78	3	41	78.85	33	63.46

ZDV-3TC-NVP	0	0	0	0	0	0	0	0	0

^a ^MSA, multiple sequence alignment

^b ^NSI, number of sequences included in the analysis (sequences having gaps and ambiguous codons were discarded)

^c ^VV, total number of viral variants (these last defined as those having nucleotide changes with respect to HXB2)

^d ^W, number of selected windows throughout the MSA, with a score threshold of 2 (windows satisfied specific requirements, see Methods)

^e ^E, entropy per window

^f ^SV, number of subdominant variants (are sequences that appear more than 4 times in an MSA, see Methods)

^g ^ST-SV, sequences targeted by the group of subdominant variants.

^h ^PC-SV, percentage of coverage by SV

^I ^ST-DV, number of sequences targeted by the dominant variant.

^j ^PC-DV, percentage of coverage by the dominant variant

**Table 3:** Best shRNAs targeting sequences in more than one MSA.

^a ^HXB2 Coordinates	^b ^shRNA Sequence	^c ^Score	^d ^Targeted MSAs	^e ^Min_ST	^f ^Max_ST	^g ^Total
**^n^**2702-2725	^h ^GCCTGAAAATCCATACAATACTCC	5	7,8,9,10	33 (7)	741 (9)	848
	GCCTGAAAATCCATA**t**AATACTCC	6.5	2,9	6 (2)	223(9)	229
	GCCTGAAAA**c**CCATACAATACTCC	5	2,9	4 (2)	62 (9)	66

^n^2333-2356	AGCAGATGATACAGTA**g**TAGAAGA	6	1,2,3,4,5,6	10 (1)	18 (6)	85
	AGCAGATGATACAGT**g**TTAGAAGA	6	1,2,3,4,5,6	23 (1)	33 (4,6)	174
	AGCAGATGATACAGTATTAGA**g**GA	3	1,2,3,4,5,6	12 (1,2)	15 (3,5)	82
	AGCAGATGATACAGTA**c**TAGAAGA	6	1,2,4,5,6,10	6 (10)	17 (4,5,6)	81
	AGCAGATGA**c**ACAGTATTAGAAGA	7	1,2,3,4,5,6	21 (1,2)	31 (3,4,5,6)	166
	AGCAGATGATACAGTATT**g**GAAGA	6	1,2,3,4,5,6	11 (1,2)	15 (3,4,5,6)	82
	**g**GCAGATGATACAGTATTAGAAGA	7	1,2,3,4,5,6	16 (1)	27 (6)	139
	^h ^AGCAGATGATACAGTATTAGAAGA	7	1,2,3,4,5,6,10	21 (10)	920 (6)	4854

^r^2556-2579	AG**t**CCTATTGA**a**ACTGTACCAGTA	2.5	9	1013 (9)	1013 (9)	1013

^r^2574-2597	CCAGTAAAATTAAA**a**CCAGGAATG	2	9, 10	60 (9)	74 (10)	208
	^h ^CCAGTAAAATTAAAGCCAGGAATG	3	9, 10	926 (9)	1267 (10)	3448
	CCAGTAAAATT**g**AAGCCAGGAATG	2	9, 10	48 (9)	77 (10)	202

^r^2702-2725	GCCTGAAAATCC**c**TACAATACTCC	3.5	9	67	67	67

^a ^Genome position according HXB2 numbering system

^b ^In lowercase, nucleotides different from HXB2 reference genome

^c ^Score is given by the accomplishment of specific sequence features

^d ^Multiple sequence alignments numbered as follows:

1. POL_DNA_**No_Recombinants**

2. GENOME_DNA_**No_Recombinants**

3. POL_DNA_**GroupM_Recombinants**

4. GENOME_DNA_**GroupM_Recombinants**

5. POL_DNA_**All_Subtypes**

6. GENOME_DNA_**All_Subtypes**

7. ZDV-3TC-ABC

8. D4T-3TC-NVP

9. ZDV-3TC-EFV

10. 2299_Resistant_Isolates

^e ^Min_ST, Minimun number of sequences targeted in an MSA. In parenthesis, the specific number of MSA, to which targeted sequences belong.

^f ^Max_ST, Maximun number of sequences targeted in an MSA. In parenthesis, the specific number of MSA, to which targeted sequences belong.

^g ^Total number of sequences targeted in all the MSAs

^h ^shRNA sequence corresponds to HXB2 reference genome

^n^shRNAs from these regions were found in non-resistant MSA, despite some of them might target resistant viral sequences.

^r^shRNAs from these regions were found in resistant MSA.

### Statistical Analyses

**Figure 2:**
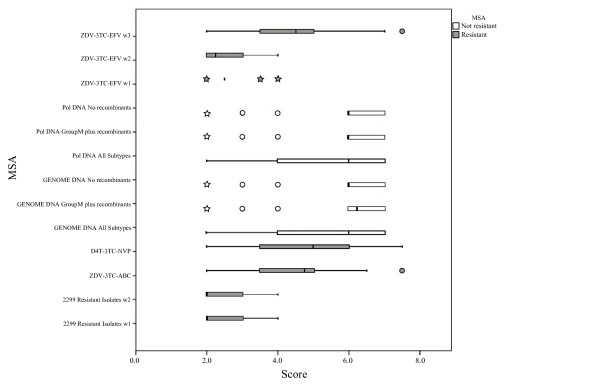
**Score Distribution among MSAs**. No scores under 2.0 are shown because this score value was the threshold used for selection by the algorithm. Circles indicate outlier values and stars indicate outlier extreme values.

**Figure 3:**
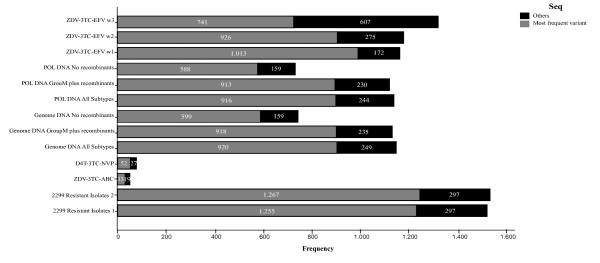
**Proportion of dominant or most frequent viral variants**. The total number of sequences is the amount of sequences that the algorithm analyzed. In the case of MSAs that have more than one window, the total number of analyzed sequences may be different. Other viral variants correspond to subdominant or totally infrequent viral sequences.

**Figure 4:**
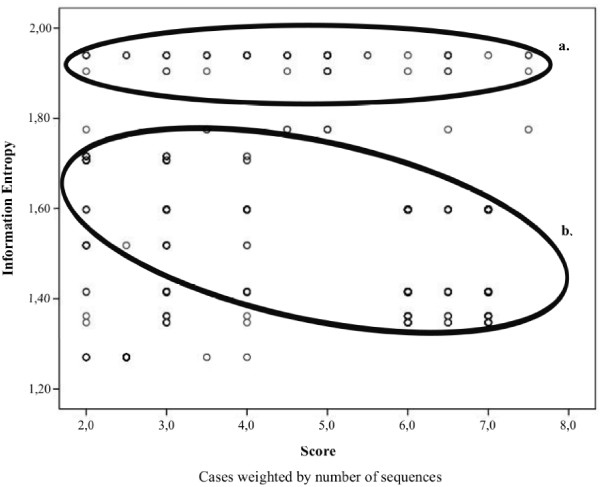
**Information Entropy and Scores correlation**. The ellipses highlight the score distribution for resistant MSAs **(a.) **and the correlation observed for non- resistant MSAs **(b.)**.

Multiple comparisons grouped non-resistant MSAs apart from resistant MSAs. There were no statistical differences (p > 0.05) within non-resistant MSAs when comparing weighted average scores, but significant differences (p < 0.05) were observed between non-resistant MSAs in comparison to resistant MSAs. In addition, there were significant differences within resistant MSAs with respect to both windows of 2299 Resistant Isolates MSA and ZDV-3TC-EFV window 2. Table 4 shows the letter code (APA) obtained for each comparison. MSAs that do have significant differences with respect to a MSAs are those whose letter code appears beneath them. In the same way they do not have significant differences with those MSAs whose letter do not appear beneath. Figure 2 is a box-plot that shows the non-symmetric distribution and atypical values of the score for each MSA. The diagram shows a clear clustering between non-resistant and resistant MSAs. Non-resistant MSAs demonstrated better scores, much higher than those obtained for resistant MSAs. Outliers and extreme values seem to make a pattern within the group of non-resistant MSAs. When comparing the proportion of sequences that can be silenced by designing shRNA against the most frequent variant, there were no significant differences (p > 0.05) within non-resistant MSAs. From resistant MSA, only ZDV-3TC-EFV_w1 MSA showed significant differences to all MSAs. Significant differences (p < 0.05) were found between both resistant and non-resistant MSAs (Table 4). Figure 3 shows the distribution of proportions of dominant and subdominant viral variants within each MSA. Entropy and Score values showed a negative, indirect and 99% significant correlation, with *r *= -0.378 (p < 0.01). Resistant MSAs which had the highest entropy values showed no preference for score values, which is in accordance with the fact that these MSAs showed much more polymorphisms than non-resistant MSAs (Figure 4).

**Table 4:** Multiple Comparisons for Score, and Proportion of dominant variants.

MSA	2299 Resistant Isolates w1	2299 Resistant Isolates w2	AZT-3TC-ABC	D4T-3TC-NVP	GENOME DNA All Subtype	GENOME DNA GroupM plus Recombinants	GENOME DNA No Recombinants	POL DNA All Subtypes	POL DNA GroupM plus Recombinants	Pol DNA No recombinants	ZDV-3TC-EFV w1	ZDV-3TC-EFV w2	ZDV-3TC-EFV w3
**Assigned letter group**	(A)	(B)	(C)	(D)	(E)	(F)	(G)	(H)	(I)	(J)	(K)	(L)	(M)

**Mean ^a^Score**			A B K L	A B K L	A B C D K L M	A B C D K L M	A B C D K L M	A B C D K L M	A B C D K L M	A B C D K L M	A B L		A B K L

**^b^0**			K	K	K	K	K	K	K	K		K	A B E F G H I J K L

**^b^1**	M	M			M	M	M	M	M	M	C D E F G H I J L M	M	

a. Weighted average of the score was used for multiple comparisons between de MSAs

b. In the comparisons of the proportion of dominant variants, number 1 represents the dominant viral variants while number 0 represents the rest of viral variants (subdominant and infrequent).

For weighted average score, a multiple comparison Student t-test was used to evaluate mean equality between each pair of groups. The MSA was assigned as the segmenting categorical variable and the score was the continuous variable for which the mean was calculated. For the comparison between pairs of proportions of dominant variants, a Z-test was used. The MSA was assigned as the segmenting categorical variable, and the proportion was assigned the categorical variable that revealed the presence or absence of the event of interest. In the second and third rows appear the corresponding letters of the groups that showed significant differences with the MSA of the column. In both cases p values were corrected with Bonferroni-Dunn test with an alpha of 0.05. See Methods, for further understanding on how weighted average scores were calculated.

### Blast

Using BLAST, eight out of forty-eight shRNAs were found in the selected databases. Results are shown in Table 3. No hit had 100% overlap, and overlap was toward the 3' terminal end of the shRNA (Additional file 3).

## Discussion

This is the first *in silico *approach to novel shRNA design based on the scored search of a group of sequences directed at silencing the dominant and subdominant most frequent wild type and mutant RT variants, targeting conserved regions. We developed an algorithm that followed previously published sequence parameters from effective shRNAs, using a free energy cut-off and specific sequence features [24,25]. No current approach targets frequent viral variants simultaneously; instead, it is usual to target several conserved regions with one sequence. The trouble is that for each of these regions, other frequent variants that do not match the reference genome sequence HXB2 need to be considered. Similar interesting works have been undertaken also analyzing publically available sequences, such as McIntyre *et al*. 2009. However, these differ from ours in that they neither searched for subdominant viral variants and/or infrequent viral variants, nor searched for shRNAs able to target resistant viruses that emerged under a specific antiretroviral selective pressure. Also, they do not describe in detail their *in silico *analyses; the features for silencing activity they evaluated, the filters or threshold they used, whether they included a free energy cut-off, their approach to ambiguities (UIPAC letter code), whether they used all the sequences, how they analyzed sequence quality in their MSAs, etc. They did design shRNAs of different lengths directed toward HXB2 reference genome, that overlaps within one of our regions -emphasizing the conservation of this part of the viral genome- however, those molecules do not match our subdominant variants. Our results identified a greater number of viral variants that any other study.

shRNA design is difficult, owing to the multiple requirements for achieving efficient silencing *in vivo*, and to all the parameters that must be carefully followed. Available programs are usually directed towards siRNA rather than shRNA design [26], and it has been shown that these programs do not always correctly predict the silencing efficiency of shRNAs [27]. Online tools do not allow for more than one aligned sequence to be used, but several aligned sequences are necessary for designing silencing molecules against error-prone viruses such as HIV. Throughout the HIV-1 genome, we identified the less variable regions that showed the best silencing predicting features. However, MSAs revealed that there is at least between 20.12% to 21.31% of naïve isolates, and between 14.51% to 45.03% -percentages result from subtracting the table values out of 100%- of resistant isolates that will not be targeted using solely the dominant viral variant (Table 2). For that reason targeting multiple genome regions with one sequence for each will not solve this problem, because each region will have different untargeted naturally occurring variants. Any design strategy based on consensus shRNA sequences is susceptible to viral escape in terms of long-term silencing, particularly in an HIV-1-infected human. HIV variability underlies the fact that key target selection is of utmost importance. The most frequent or dominant shRNA (one sequence) in all the alignments fell between 63.46% and 85.49% of the viral sequences with an average value of 75.20% (Table 2). This is consistent with previous findings in which targeting a single region resulted in rapid emergence of resistance by means of selecting subdominant variants -those that remained untargeted [28]. Achieving a higher silencing could be obtained by targeting subdominant variants from the same region like the subdominant variants we found (Table 3 and Additional file 1. Ideally, all the viable changes in each targeted conserved sequence must also be targeted in order to achieve life-long silencing. For this we first attempted to analyze further viral variability on the basis of protein function or biological significance, which is thought to show the lowest variability. From the selected regions based on protein function, only region number 2 of RT conserved domain provided results (Table 1 and Table 3). This was probably because we were not merely looking for a conserved region, but a conserved region that met specific requirements such as free energy values and sequence specific features. This was based on the fact that shRNAs that are perfectly matched with their target sequences do not necessarily achieve 100% silencing. Nonetheless, our shRNAs targeted only two regions in PR and one in RT, highlighting the conservation of these regions despite analyzing complete genome sequences; complete genomes provided the same windows. It is interesting that all the HIV-1 group M sequences behave within the same limits of variability, and the inclusion of recombinants did not affect the results. High scores were predominant in these sequences, implying that within the selected regions changes are allowed preferably in the same positions, not randomly. Highest scores were not reached; this means that intrinsic HIV-1 sequence characteristics and variability are an obstacle to expecting specific silencing sequence features in shRNA molecules. In fact, reaching the highest score demands for a highly conserved region in which changes are limited to certain positions and certain nucleotide changes. The latter is due to the fact that there are multiple sequence features that need to be satisfied throughout the silencing molecule in such a way that increasing variability would reduce the probability of achieving them. Differences were only significant when analyzing resistant MSAs. Low scores of these sequences are attributable to the degree of polymorphisms that seem not to have any pattern, and to drug selected mutations. Changes can occur almost in any place of the 23 nt window with differences in frequency per position, but with no apparent restriction. That's why resistant MSA showed the highest entropy values with the lowest scores. Recently, Schopman et al. [29] showed that targeting common resistant variants that emerge under silencing therapy decreased viral escape, but then new routes of evading silencing were used by the virus. This is explained by our analyses, which showed that there is over 20% variability that the virus can use to escape, without any selective pressure (non-resistant MSAs). Resistant MSAs showed the capability of the virus to mutate much further beyond this 20%. In fact, non-resistant MSAs were grouped together and apart from resistant-MSAs (Table 4.). Window 1 from ZDV-3TC-EFV was different from all the other MSA (resistant and non-resistant) in dominant viral variants, and W3 from the same MSA was different also in subdominant viral variants. These results are consistent with the fact that W1 dominant viral variant is different from HXB2 reference sequence and also with the fact that W3 had the lowest entropy value, which is the same as saying that it showed the highest variability. Resistant-MSAs constitute an insight to understand virus evolution; nonetheless we doubt those to show the true limits. In any case, targeting the dominant and subdominant viral variants for each region may reduce this set of viable changes.

We did not find any other genome region to be targeted, probably due to some of the parameters used such as "number of sequences" in which regions that are not well represented by a certain number of sequences are discarded. Another reason is that other stringent conditions besides sequence conservation were assessed. Unfortunately, genome ends are underrepresented, which leaves long terminal repeats (LTRs) and other terminal regions outside of the study. LTR is thought to be a good region for this type of strategy, but the variability of this region cannot be addressed accurately due to the relative small number of complete sequences present in the databases. There is another explanation for not having found shRNAs for key regions within the RT conserved domain. For example, the conserved nucleotide positions for the YMDD motif ranges from 1 to 8 out of 12, in the nucleotide reference MSA from the *pol *gene (Los Alamos HIV Databases). The amino acid reference sequence for the window with the fewest variants was WPLTEEK, which can be formed by 512 different nucleotide sequences. The mutations throughout the reference Pol polyprotein MSA (Los Alamos HIV Databases) are W24R, P25LTS, T26SA, E27KAGR, E28K and K29ER, and these collectively give 286,654,464 possible nucleotide combinations. Another reason could lie in the three nearby amino acids (either to the left or to the right of the motif), which can be encoded by more than two codons due to the redundancy of the genetic code.

**Figure 5:**
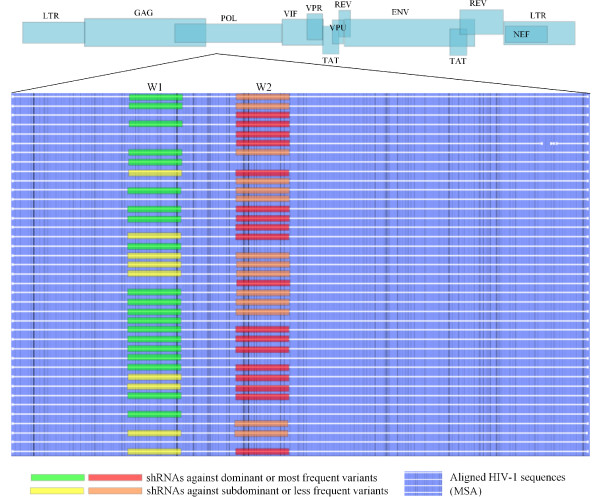
**Silencing Model**. Targeting dominant variants from two or more regions leaves several subdominant viral variants untargeted. The optimal approach would be a cocktail of carefully selected molecules targeting dominant as well as subdominant variants from more than one conserved region. The figure shows a schematic representation of HIV-1 genome and an MSA of HIV-1 *pol *gene, in which the strategy of silencing is drawn. Some sequences would be targeted by two shRNAs, some just by one, and a few would not be targeted at all, but are not frequent. W1 and W2 represent the hypothetical targeted regions, where "W" stands for "window".

Altogether, our group of shRNAs might be able to silence at least 94% of the sequences present in the alignments, just by perfect matching. This means that it is possible to target almost every virus at least once, with a selective group of shRNAs. Untargeted sequences can probably be targeted including frequent shRNAs from a different region, as is shown in Figure 5. Though it must be considered that an uncommon sequence variant either was the dominant one in a patient, or was the amplified quiasispecie, or it could have also been a sequencing error. Since evolution depends on time, intrapatient viral evolution can turn rare variants into dominant ones, so the selection of frequency threshold could not be picked too high. Because of this, sequences that appeared 4 or more times in an alignment were named frequent sequences. Frequent variants -including both dominant and subdominant- usually have higher fitness, so rare variants may be less pathogenic and perhaps controllable by the host immune system. shRNAs found in this study have high silencing scores, meet the energy threshold needed for efficient loading into RISC complex, and target most of the viral sequences analyzed *in silico*. Free energy threshold is fundamental for guide strand selection and mounting into the RISC complex, increasing the silencing efficiency of our molecules. Off-target effects against *Homo sapiens *protein phosphatase 2C, mRNA, should be tested *in vitro*. This is done in order to address the real silencing of the genes when using the set of shRNAs that have 17 bp of identity with this human gene, 15 of which are directly related with silencing (two of them correspond to 3´ terminal overhangs). shRNAs with 20/20 identities with a BLAST hit should be avoided, unless proven to be safe.

In 2004, an siRNA against *nef *was used to inhibit HIV-1 *in vitro*, however in these assays, several weeks later escape mutant viruses emerged. That was one of the first studies to propose that virus variability should be addressed with a combination of siRNAs [30] Escape happened because the selected target sequence is biologically dispensable, a fact that should not be ignored even though the target was not thought to be *nef *itself, but all the RNA viral variants which are supposed to have this sequence. Now this underlines the importance of including much more biological information criteria for target selection than simply sequence conservation or the presence of a sequence in multiple viral transcripts. In fact, we found no conservation in any of the 21 nucleotides of the *nef *sequence used, throughout an MSA (data not shown). In other words, silencing was directed against an uncommon or subdominant variant of the virus, a fact that could have played a role in resistance development. However, it is important to highlight that HIV-1 silencing has been achieved in *in vivo *mouse models. Silencing was observed in a mouse model systemically infected with a combination of siRNAs consisting of a human specific CCR5 siRNA and two viral specific siRNAs [15], and no evidence of viral escape mutants was observed. Furthermore, a mouse model was engrafted with human hematopoietic stem cells that were previously transduced with a lentiviral vector carrying a shRNA against *nef*. While transduction efficiency in this *in vivo *model was reduced, *ex vivo *transduction of mature CD4 (+) cells lead to wild type HIV-1 resistant T cells in vivo [20]. Now, even though certain studies have shown the emergence of resistant variants, our results raise the question whether they always result from "resistance" to RNAi therapy or whether they are naturally occurring uncommon variants that are being selected rather than targeted. So even though there was no evidence of resistance in the first mouse model--and despite the fact it was clearly shown that the silencing effect is sequence specific, regardless of the type of infection used (systemic or *ex vivo*) and regardless of the time an experiment is assessed--it cannot be said for sure that resistance wouldn't have occurred later. Even when assessing resistance was not the aim of these studies, it constitutes a threat for therapy outcome. In fact, resistance against HAART inhibitors arises in many human cases within a few years of initiating therapy, usually 2-4, depending on different variables [7,31-34]. Then, longer periods of time in an animal model are therefore needed to evaluate the emergence of resistance or the inhibition of virus replication, especially since treatment has to be life-long. Our target was RT because it cannot be deleted from the genome and is the main source of virus variability. Since it is a HAART target, information about mutations induced under selective drug pressure is available [33,35-38]. Reverse transcription is a prerequisite for viral integration and infection of other cells, so its silencing would reduce the number of cells that become actively and latently infected, preventing the establishment of more HIV cell reservoirs.

Several groups have also designed silencing molecules within our same *pol *region, confirming the importance of this region as an HIV target [16,19,21,23,30,39,40]. However, these previous molecules do not exactly begin from the same position as ours, have different lengths, and are only directed to silence viruses that match the dominant HXB2 reference genome sequence or specific mutant viruses that emerge during RNAi experiments in prolonged cell culture. We compared our dominant shRNAs to 100 previously published *pol*-directed silencing molecules and none of them was found to be identical, but some do map really near within the virus genome (data not shown). The nearest previous published molecule (2328-2346 with respect to HXB2) [19] maps 5 nt downstream of our targets against non-resistant isolates (2333-2356 with respect to HXB2). Seven other molecules were designed from position 2326 to 2360 with respect to HXB2 [23], and three more targets were designed in region 4752 to 4775 [23], overlapping our shRNA for non-resistant isolates that was identified when analyzed with no score restriction. Our molecule mapped within position 4747- 4770 with respect to HXB2 (Additional file 2). There are neither previous reports concerning shRNAs targeting viruses resistant to current first line antiretrovirals, nor reports about shRNAs targeting other dominant or subdominant viral variants within the same genome region. To target viruses resistant to certain antiretrovirals or to certain line of antiretrovirals with RNAi while taking them, or to target dominant and subdominant viral variants simultaneously, may hypothetically impede viral escape due to a major reduction in the possible available nucleotide changes that are not deleterious to the virus. This type of approach might cover many more viral variants than using just one strategy, but it will not solve economic issues and side effects concerning life-long HAART.

## Conclusions

The emergence of resistant viral variants is an inconvenience that must be addressed carefully, particularly in the case of using shRNAs since they normally work in a sequence-specific manner. In this study we identified dominant and subdominant frequent viral variants representing the set of naturally occurring changes that are observed in the viral population that has been sequenced world-wide, and we designed shRNAs against these. We found that in order to cover all the viral variability to impede viral escape, we will need to use too many silencing molecules, which is not clinically feasible. Even in the absence of a selective pressure such as antiretrovirals, there are plenty of polymorphisms that can occur throughout the viral genome that can allow the virus to escape RNAi therapy. In addition, drug selective pressure is capable of inducing even more unusual changes. Although it is difficult to determine the exact mechanism by which it is possible to completely avoid viral escape, what is important now is that we have identified most of what we have to set upon. Our shRNA cocktail was developed based on the viral variability we found, and there is chance that it could be used alone (cocktail) or as a complement to HAART. Several authors have also proposed a cocktail of shRNAs to target HIV-1, but our cocktail is different in that it was designed to target dominant as well as subdominant viral variants. Long-lasting silencing for humans seems much more possible with this complete approach. Our results point towards the conclusion that viral population is modeled by selective pressures, since it was possible to find shRNAs for most of the regimens (it was, however, difficult for ZDV-3TC-NVP). Special attention must be taken regarding the fact that selective pressures, including silencing molecules, may induce or push the virus to mutate within the limits of resistance to antiretrovirals, since most changes are non- deleterious. Further studies are needed to find regimens with resistance patterns that can be specified and better controlled using the fewest number of shRNAs. Proving real *in vivo *efficiency in combination therapy, as well as identifying off-target and non-specific off-target effects of the shRNAs, is absolutely required before starting any therapeutic approach.

Our study is the first in identifying naturally occurring and induced nucleotide changes in the virus, based on data from viruses modeled by natural selection from natural hosts (humans) and from viruses modeled by drug selective pressure in treated patients.

This work is important to understanding the complexity of HIV-1 variability, in order to be able to target it effectively. Indeed, nucleotide substitutions have occurred under RNAi selective pressure, but we have shown in this study that there are much more nucleotide changes that can occur which may result in viral escape. Different approaches might work while trying to address viral ability to escape RNAi therapy, but undoubtedly the more information we know about the mechanisms the virus uses to do so, the more we can do about it.

## Methods

### Conserved regions and prevalent drug-resistant mutations: target sites

Pol polyprotein AAB50259.1 from the HXB2 reference sequence was used as query against the conserved domain database (CDD) of NCBI [41] in order to identify catalytic residues, residues involved in DNA binding, dNTP binding, or active site residues with no other annotation. The map of coordinates from the HXB2 reference sequence accession number K03455 (gi|1906382|gb|K03455.1|HIVHXB2CG human immunodeficiency virus type 1 (HXB2), complete genome; HIV1/HTLV-III/LAV reference genome), available at Los Alamos HIV databases http://www.hiv.lanl.gov, was used to find the exact positions of selected regions in MSAs using the sequence as a guide. The complete genomes were also assessed in order to identify other possible targets within conserved regions outside of the *pol *gene.

In addition, high prevalence resistance mutations were selected from the "Mutation Prevalence According to Subtype" web page http://hivdb.stanford.edu/cgi-bin/MutPrevBySubtypeRx.cgi from the Stanford HIV Drug Resistance Database (HIVRT&PrDB) http://hivdb.stanford.edu/[42]. Mutations which were located within the selected regions of the conserved domain were preferred.

### Sequence retrieval and MSAs

Sequences from resistant isolates were retrieved from HIVRT&PrDB by a therapy criterion that consisted of different combinations of three antiretrovirals, two nucleoside analog reverse transcriptase inhibitors (NRTI) and one non-nucleoside analog reverse transcriptase inhibitor (NNRTI). These sequences were not grouped by a specific regimen; instead they were grouped together as sequences from resistant viruses that emerged under different first line regimens. Duplicates and repeated sequences were eliminated using the Elim Dupes tool http://www.hiv.lanl.gov/content/sequence/ELIMDUPES/elimdupes.html from Los Alamos HIV databases. Sequences were further processed to eliminate foreign characters not compatible with FASTA format (e. p. "~"), and were then aligned to generate an MSA. In addition, first-line regimens that are used in Colombia were selected from the "National Resolution No. 3442-2006 for the care of HIV-infected patients", approved for treatment of HIV infection in Colombia by the Ministerio de Protección Social [43]. This was done in order to retrieve sequences generated by the specific drug-selective pressure induced by each regimen in our country. The regimens were zidovudine-lamivudine-efavirenz (ZDV-3TC-EFV), which is normally the first choice, zidovudine-lamivudine-nevirapine (ZDV-3TC-NVP), zidovudine-lamivudine-abacavir (ZDV-3TC-ABC) and stavudine-lamivudine-nevirapine (D4T-3TC-NVP), which are also first line regimens. All of these select M184V mutation. Sequences were aligned by HMM (Hidden Markov Models) model HIV-1/SICcpz [44] into four independent MSA, one for each regimen. MSAs were generated using the HIVAlign tool from Los Alamos HIV databases, which is an implementation of the HMMER package http://  http://www.hiv.lanl.gov/content/sequence/HMM/HmmAlign.html, and sequences were codon-aligned with Gene Cutter http://www.hiv.lanl.gov/content/sequence/GENE_CUTTER/cutter.html. HXB2 was included in each MSA to identify the selected regions. MSAs were further edited manually using MSA editors Bioedit and eBIOX, and analyzed in CLC DNA Workbench http://www.clcbio.com (Aarhus, Denmark).

Sequences from naïve isolates were retrieved from Los Alamos HIV databases. MSAs were generated as mentioned and downloaded from this database. In order to address the impact of virus variability in the aim of the study, different type of MSAs were downloaded from the database (i.e. Subtype specific, *pol *gene specific, with and without recombinants, complete genomes, all subtypes, whole group M). HXB2 was also included in each alignment and MSAs were also manually edited.

Additionally, 35 Colombian isolates from symptomatic HIV-positive patients were included in the study, and the fasta sequences were incorporated into the MSA of the non-regimen-specific resistant viral sequences. Only samples with written informed consent, complete clinical history and viral loads over 1000 copies/ml were included. RNA was extracted using the semi-automated Nuclisens Minimag and Nuclisens Extraction Kit according to the manufacturer's instructions (Biomerieux, Marcy l'Etoile, France). HIV was genotyped with a commercial TRUGENE HIV-1 kit (Siemens Diagnostics, Deerfield, USA), and fasta sequences were further analyzed with HIVdb software from HIVRT&PrDB. Quality assessment and Calibration Population Resistant (CPR) tools were used to evaluate probable sequencing errors and mutation prevalence, respectively.

### Variability analysis and shRNA design

A Bioperl-based algorithm was developed to identify nucleotide changes throughout the multiple MSAs. The algorithm was developed to identify all the existing viral sequence variants and their frequencies throughout an MSA, while evaluating their silencing efficiency based on free energy and entropy calculations, in addition to a score that represented how many specific sequence features they met. The HIV genomes were processed in order to extract potentially useful sequences for the synthesis of shRNA

$$Hp=-\sum_{i=0}^{n}\pi_{i}*log(\pi_{i})$$

molecules. The genomes were first aligned using HMM (see sequence retrieval and MSAs) and the MSAs were scanned using sliding windows of 23 bp. For each window, the information entropy as defined by Shannon [45] was calculated:

Where *n *is the number of different sequences within each window, π_i _is the proportion of the *i*-th sequence (*i.e*. the number of times this sequence appears in the window divided by the number of sequences in the alignment), and *H_p _*is the information entropy of the *p*-th window.

Only windows with information entropy below two -allowing viral variability to be studied- and with more than 40% of informative sequences (*i.e*. not entirely composed by gaps) were accepted, avoiding windows that were too variable, and two sets of conditions were evaluated on every different sequence of the window. First, we evaluated mandatory conditions, accepting only sequences:

• With less than 10% gaps,

• No gaps in the middle of the sequence,

• No ambiguous characters (*i.e*. only A, C, T and G accepted),

• No G or C stretches of 4 bp or more in length,

• No T stretches of 3 bp or more in length,

• Starting with T or C,

• With GC percentage between 30 and 50,

• ΔG measured in the 5' last 7 bp of the sense sequence above -6.6, and

• ΔG measured in the 5' last 6 bp of the antisense sequence above -6.6.

The ΔG were calculated as described by Freier *et al *[46]. Afterwards, we evaluated a score only on accepted sequences, defined as a sum of differentially scored properties

• The sequence starts with AA, AT, TT or TA: +0.5,

• The sequence ends with AA, AT, TT or TA: +0.5,

• The AT count of the 15-21 position range is at least 4: +1, or more than 4 (+1 for every additional A or T in the range),

• The 21st position is A or T: +1,

• The 5th position is C or G: +0.5,

• The 7th position is G: +0.5,

• The 13th position is A or T: +0.5,

• The 16th position is T: +0.5,

• The 8th position is C and the 9th position is A: +1,

• The 10th and 11th positions are C: +1,

• The 12th position is T and the 13th position is G: +1,

• The 15th position is G and the 16th position is A: +1,

• The 19th position is T and the 20th position is A: +1,

• The 20th position is T and the 21st position is A: +1,

• The GC count is below 11: +1,

• The 21st position is G or C: -1,

• The 5th position is T: -0.5,

• The 6th position is T and the 7th position is A: -1,

• The 8th position is T and the 9th position is A: -1,

• The 10th and the 11th positions are G: -1,

• The 11th position is G and the 12th position is C: -1, and

• The 19th position is G and the 20th position is A: -1

Unique sequences with a score equal or greater than two were accepted because they had at least two efficient sequence specific silencing features, an advantage over silencing molecules that are made to follow only a sequence specific silencing; higher score thresholds would have resulted in the elimination of too many sequences per window leaving less than an 80% of the initial amount, rendering further analyses not worthy. Only windows with at least 80% of sequences accepted were used in further analyses. Within each accepted window, sequences that appeared 4 or more times were named as subdominant viral variants, viral variants meaning different from reference HXB2 sequence. No statistical approach was undertaken to asses this frequency limit, it was chosen in order to avoid accepting nucleotide changes that could have been sequencing errors, but at same time trying to rescue very infrequent but viable and possible ones accepted within the limits of virus evolution. Dominant viral variant, refers to the viral variant that appears the most, but in this case it may be exactly the same as reference HXB2 sequence. A window might have one or more dominant variants when there are sequences that appear a similar number of times as the dominant viral variant in a MSA. All the accepted sequences in the accepted windows were searched against the NCBI's Human RNA database using BLAST [47] with an e-value threshold of 0.1. Sequences were also searched against the Human genomic sequences in order to search for a potentially transcribed sequence not identified so far as mRNA, with identical parameters.

**Figure 6:**
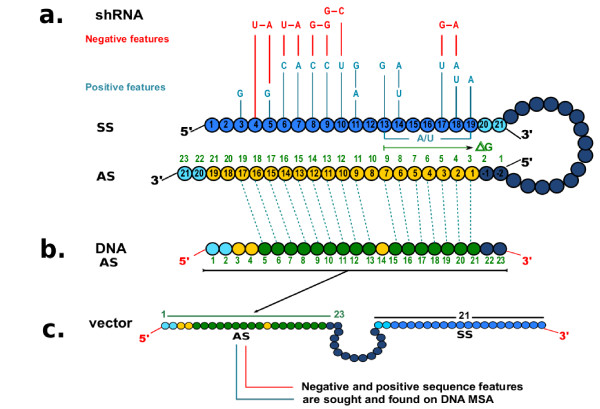
**shRNA diagram**. **(a) **Schematic representation of shRNA showing important sequence features with corresponding positions in the antisense strand. **(b) **DNA antisense strand indicating the correction of positions with respect to antisense strand from shRNA **(c) **DNA vector (only shRNA is shown, partial sequence). Correction of sequence features positioning is fundamental for seeking them in MSA with the algorithm, since MSA are DNA sequences.

The algorithm evaluated the predicted efficiency of silencing in the DNA viral sequence to be transcribed as a guide strand (Figure 6). The algorithm source code is freely available online via GPL license in order to be used and improved by others, at http://bioinf-mac.uniandes.edu.co/shrna

### Statistical Analyses

Data was organized considering each MSA as an independent group, and statistical analyses were performed with SPSS for windows package V 8.0.0 (SPSS Inc., IBM, Chicago, Illinois). A descriptive analysis was performed to describe quantitative variables for which weighted average values were calculated, and qualitative variables were expressed as frequencies. For all the multiple comparisons, the non parametric Bonferroni-Dunn correction test was used. Weighted average scores obtained for each MSA were compared in order to address if scores were significantly different within all categories using a Student t-test (p < 0.05, α = 0.05). These weighted average scores were calculated as the average score for each MSA, but it was weighted with respect to the number of sequences that showed that score. This is why it is a "weighted" average score. The Spearman correlation coefficient was used to address Score-Entropy non-causal association (p < 0.01, α = 0.01). Differences in the proportion of sequences that could be targeted by the most frequent variant of each MSA were also addressed with a Z-test (p < 0.05, α = 0.05). Comparisons were made with no discrimination between resistant and non-resistant viral sequences. For MSAs for which more than one window was selected by the algorithm, each window was treated independently.

## Competing interests

The authors declare that they have no competing interests.

## Authors' contributions

MCMO developed the idea, worked out the results, interpreted and analyzed the data, wrote the manuscript and edited all the images. JCMS developed the initial script, which evaluated sequence features in multiply-aligned sequences and developed other tools for editing special characters from sequences. LMR-R developed the final algorithm, which included the free energy filter, modifiable selection thresholds, information entropy calculation and the possibility of scanning whole genomes in an automatic way. IP made the statistical analyzes and helped in their interpretation. SR, APM, RS and GJRB critically reviewed the manuscript. All authors read and approved the final manuscript.

## Supplementary Material

Additional file 1 **Frequent shRNAs in all the MSA**. The table shows all the frequent shRNAs (48), each of which occurred at least 4 times in an alignment. Click here for file

Additional file 2 **Data File**. There are two sheets that correspond to raw data. The first sheet has the results obtained using a score threshold of 2, and the other sheet has the results obtained with no score threshold. Notice that in this second sheet, the same windows appeared that were selected in the first sheet, in addition to new windows that were initially discarded due to score threshold. All these new windows passed the first filters in which free energy calculation is included. Click here for file

Additional file 3 **BLAST Hits Description**. Complete information for each BLAST hit found in the databases is described. Click here for file
