# Supplementary material for: An RNAi in silico approach to find an optimal shRNA cocktail against HIV-1
Source: Virol J. 2010 Dec 20;7:369. doi: 10.1186/1743-422X-7-369 (PMC3022682; doi:10.1186/1743-422X-7-369)
Supplement: Additional file 3 — BLAST Hits Description. Complete information for each BLAST hit found in the databases is described. [file 1743-422X-7-369-S3.DOC]

**Additional File 3. Blast Hits Description**

**Reference mRNA sequences (refseq_rna Database)**

>ref|NM_018444.2| Homo sapiens protein phosphatase 2C, magnesium-dependent, catalytic subunit (PPM2C), nuclear gene encoding mitochondrial protein, mRNA

>ref|NM_014021.2| Homo sapiens synovial sarcoma, X breakpoint 2 interacting protein, (SSX2IP), mRNA

>ref|NM_198679.1| Homo sapiens Rap guanine nucleotide exchange factor (GEF) 1, (RAPGEF1), transcript variant 2, mRNA

>ref|NM_005312.2| Homo sapiens Rap guanine nucleotide exchange factor (GEF) 1, (RAPGEF1), transcript variant 1, mRNA

**Human Genomic Sequence Database**

>ref|AC_000135.1|AC_000135 Homo sapiens chromosome 3, alternate assembly (based on HuRef), whole genome shotgun sequence

>ref|AC_000046.1|AC_000046 Homo sapiens chromosome 3, alternate assembly (based on Celera assembly), whole genome shotgun sequence

>ref|NC_000003.10|NC_000003 Homo sapiens chromosome 3, reference assembly, complete sequence

>ref|AC_000133.1|AC_000133 Homo sapiens chromosome 1, alternate assembly (based on HuRef), whole genom shotgun sequence

>ref|NC_000001.9|NC_000001 Homo sapiens chromosome 1, reference assembly, complete sequence

>ref|AC_000044.1|AC_000044 Homo sapiens chromosome 1, alternate assembly (based on Celera assembly), whole genome shotgun sequence
